# ^18^F-FPYBF-2, a new F-18-labelled amyloid imaging PET tracer: first experience in 61 volunteers and 55 patients with dementia

**DOI:** 10.1007/s12149-018-1236-1

**Published:** 2018-01-31

**Authors:** Tatsuya Higashi, Ryuichi Nishii, Shinya Kagawa, Yoshihiko Kishibe, Masaaki Takahashi, Tomoko Okina, Norio Suzuki, Hiroshi Hasegawa, Yasuhiro Nagahama, Koichi Ishizu, Naoya Oishi, Hiroyuki Kimura, Hiroyuki Watanabe, Masahiro Ono, Hideo Saji, Hiroshi Yamauchi

**Affiliations:** 1 Shiga Medical Center Research Institute, Moriyama, Japan; 2 Department of Molecular Imaging and Theranostics, National Institute of Radiological Sciences (NIRS), National Institutes for Quantum and Radiological Science and Technology (QST), Chiba, Japan; 3 Department of Geriatric Medicine, Shiga General Hospital, Moriyama, Japan; 4 Kawasaki Memorial Hospital, Kawasaki, Japan; 5 Human Health Sciences, Graduate School of Medicine, Kyoto University, Kyoto, Japan; 6 Research and Educational Unit of Leaders for Integrated Medical System, Center for the Promotion of Interdisciplinary Education and Research, Kyoto University, Kyoto, Japan; 7 Department of Analytical and Bioinorganic Chemistry, Kyoto Pharmaceutical University, Kyoto, Japan; 8 Department of Patho-Functional Bioanalysis, Graduate School of Pharmaceutical Sciences, Kyoto University, Kyoto, Japan

**Keywords:** Alzheimer disease, Amyloid imaging, Healthy volunteers, Positron emission tomography

## Abstract

**Objective:**

Recently, we developed a benzofuran derivative for the imaging of β-amyloid plaques, 5-(5-(2-(2-(2-^18^F-fluoroethoxy)ethoxy)ethoxy)benzofuran-2-yl)-*N*-methylpyridin-2-amine (^18^F-FPYBF-2) (Ono et al., J Med Chem 54:2971–9, 2011). The aim of this study was to assess the feasibility of ^18^F-FPYBF-2 as an amyloid imaging PET tracer in a first clinical study with healthy volunteers and patients with various dementia and in comparative dual tracer study using ^11^C-Pittsburgh Compound B (^11^C-PiB).

**Methods:**

61 healthy volunteers (age: 53.7 ± 13.1 years old; 19 male and 42 female; age range 24–79) and 55 patients with suspected dementia [Alzheimer’s Disease (AD); early AD: *n* = 19 and moderate stage AD: *n* = 8, other dementia: *n* = 9, mild cognitive impairment (MCI): *n* = 16, cognitively normal: *n* = 3] for first clinical study underwent static head PET/CT scan using ^18^F^−^FPYBF-2 at 50–70 min after injection. 13 volunteers and 14 patients also underwent dynamic PET scan at 0–50 min at the same instant. 16 subjects (volunteers: *n* = 5, patients with dementia: *n* = 11) (age: 66.3 ± 14.2 years old; 10 males and 6 females) were evaluated for comparative study (50–70 min after injection) using ^18^F-FPYBF-2 and ^11^C-PiB on separate days, respectively. Quantitative analysis of mean cortical uptake was calculated using Mean Cortical Index of SUVR (standardized uptake value ratio) based on the established method for ^11^C-PiB analysis using cerebellar cortex as control.

**Results:**

Studies with healthy volunteers showed that ^18^F-FPYBF-2 uptake was mainly observed in cerebral white matter and that average Mean Cortical Index at 50–70 min was low and stable (1.066 ± 0.069) basically independent from age or gender. In patients with AD, ^18^F-FPYBF-2 uptake was observed both in cerebral white and gray matter, and Mean Cortical Index was significantly higher (early AD: 1.288 ± 0.134, moderate AD: 1.342 ± 0.191) than those of volunteers and other dementia (1.018 ± 0.057). In comparative study, the results of ^18^F-FPYBF-2 PET/CT were comparable with those of ^11^C-PiB, and the Mean Cortical Index (^18^F-FPYBF-2: 1.173 ± 0.215; ^11^C-PiB: 1.435 ± 0.474) showed direct proportional relationship with each other (*p* < 0.0001).

**Conclusions:**

Our first clinical study suggest that ^18^F-FPYBF-2 is a useful PET tracer for the evaluation of β-amyloid deposition and that quantitative analysis of Mean Cortical Index of SUVR is a reliable diagnostic tool for the diagnosis of AD.

**Electronic supplementary material:**

The online version of this article (10.1007/s12149-018-1236-1) contains supplementary material, which is available to authorized users.

## Introduction

Alzheimer’s disease (AD) is the most common neurodegenerative disorder and the most common cause of dementia in the elderly, which affects 47 million patients worldwide with steadily increasing numbers [1]. The two characteristic neuropathological changes observed in AD are the deposition of extracellular amyloid senile plaques and the presence of intracellular neurofibrillary tangles (NFTs) [2, 3]. The amyloid cascade hypothesis has been proposed and the deposition of amyloid beta (Aβ) protein is the first step of Alzheimer’s pathology including NFTs [4]. Therefore, the deposition of Aβ protein has been the main target of in vivo diagnostic imaging tool of AD. Several imaging tracers, especially for positron emission tomography (PET), has been developed and reported to evaluate amyloid deposition, such as ^11^C-Pittsburgh compound B (PiB) [5], ^11^C-BF227 [6], ^18^F-AZD4694 [7], ^18^F-FACT [8], ^18^F-BAY-949172 (^18^F-florbetaben) [9], ^18^F-AV-45 (^18^F-florbetapir) [10], and ^18^F-GE067 (^18^F-Flutemetamol) [11]. PiB, the first amyloid imaging PET tracer, has been reported with successful results and used widely as a research tool [12]. However, the short half-life of labelled ^11^C (20 min) limits the clinical utility of ^11^C-PiB as a diagnostic tracer. Therefore, several ^18^F-labelled beta amyloid tracers have been developed for commercial utility because ^18^F with half-life 110 min has been recognized as commercially available radioactive tracer for clinical practice in terms of cost, supply and shipping.

Recently, we developed a benzofuran derivative for the imaging of Aβ protein, 5-(5-(2-(2-(2-^18^F-fluoroethoxy)ethoxy)ethoxy)benzofuran-2-yl)-*N*-methylpyridin-2-amine (^18^F-FPYBF-2) [13]. This new fluorinated benzofuran derivative, which is like ^18^F-AZD4694 but has a fluoropolyethylene glycol side chain, is a promising PET probe for cerebral Aβ plaques imaging, and the specific labeling of Aβ plaques was observed in autoradiographic sections of autopsied AD brain. It should be noted that ^18^F-FPYBF-2 has a stable chemical structure which does not photodegrade. However, there has been no report evaluating the utility of ^18^F-FPYBF-2 as a PET tracer in in vivo human study.

The aim of this study was to assess the feasibility of ^18^F-FPYBF-2 as an amyloid imaging PET tracer in a first clinical study with healthy volunteers and in comparative dual tracer study using ^11^C-PiB, and to evaluate the clinical usefulness of ^18^F-FPYBF-2 PET/CT in the diagnosis of AD.

## Materials and methods

### Healthy volunteers

From March 2013 to July 2014, 61 healthy volunteers (male: 19, female: 42; mean age: 53.7 ± 13.1; age range: 24–79) (Table 1) were included and underwent ^18^F-FPYBF-2 PET or ^18^F-FPYBF-2 PET/CT study as a first clinical study. Eligibility criteria for healthy volunteers (20 years old or older) in the present study were as follows; (1) who did not give any subjective complaint about cognitive problem, and (2.1) who made a declaration of their healthy status without medication, or (2.2) who had underlying non- neurological illness, such as hypertension, diabetes, hyperlipidemia, but controlled them well by medication as an out-patient-based medical practice. Exclusion criteria were as follows; (1) who had a subjective complaint or objective symptom of cognitive problem, (2) who were treated with or had past history of neurological disorder and related diseases, (3) who were treated with or had past history of brain or head injury. Each volunteer gave a written informed consent form defined by our institutional review boards with the information about the expected radiation exposure. After obtaining the written informed consent, mini-mental state examination (MMSE) was performed in each volunteer. The tracer study for healthy volunteers was approved by our institutional review boards, the Human Study Committee (approved on Mar. 28, 2013) and the Committee for the Clinical Use of Short Half Life Radioactive Materials (approved on Mar. 1, 2013), where our protocol was investigated according to the results of animal studies of safety performed in 2012 as an extended single intravenous dose toxicity study, which was based on the protocol of Guidance for the Performing of Microdose Clinical Trials announced by the Ministry of Health, Labour and Welfare of Japan.

**Table 1:** Characteristics of total 61 healthy volunteers

Gender, *n* (%)		Age (years)	Age range	MMSE	
Male	*n* = 19 (31%)	55.6 ± 13.6	24–73	28.9 ± 1.4	n.s
Female	*n* = 42 (69%)	53.4 ± 13.5	37–79	29.1 ± 1.5	n.s
Total	*n* = 61	53.7 ± 13.1	24–79	29.1 ± 1.5	

MMSE Analysis in Age (years old)			MMSE Range	MMSE	
20–39	*n* = 8		29–30	29.9 ± 0.4	n.s
40–49	*n* = 18		26–30	29.3 ± 1.4	n.s
50–59	*n* = 16		26–30	29.1 ± 1.5	n.s
60–69	*n* = 7		26–30	29.3 ± 1.5	n.s
70–79	*n* = 12		25–30	28.1 ± 1.8	n.s

Mean Cortical Index Analysis (years old)	50–60 min (1)	60–70 min (2)	% Increase (1) to (2)	50–70 min (average)	
20–39	1.016 ± 0.046	1.032 ± 0.045	1.6	1.024 ± 0.045	*p < 0.05
40–49	1.050 ± 0.053	1.069 ± 0.053	1.8	1.059 ± 0.053	n.s
50–59	1.053 ± 0.063	1.071 ± 0.060	1.8	1.062 ± 0.064	n.s
60–69	1.066 ± 0.085	1.091 ± 0.098	2.3	1.078 ± 0.091	n.s
70–79	1.087 ± 0.091	1.105 ± 0.096	1.6	1.096 ± 0.093	*p < 0.05
Total	1.056 ± 0.068	1.075 ± 0.071	1.8	1.066 ± 0.069	

Mean Cortical Index Analysis	50–60 min (1)	60–70 min (2)	% Increase (1) to (2)	50–70 min (average)	
Male	1.035 ± 0.064	1.054 ± 0.065	1.9	1.044 ± 0.064	n.s
Female	1.066 ± 0.068	1.085 ± 0.072	1.7	1.076 ± 0.070	n.s

*MMSE* mini mental state examination, *SUVR* standardized uptake value ratio

* Correlation between SUVR of the group of 20–39 and that of 70–79 years old

### Patients

From Oct. 2013 to Jul. 2016, 55 patients (male: 29, female: 26; mean age: 74.4 ± 9.4) (Table 2) who consulted our out-patient clinic of Dept. of Geriatric Medicine and were suspected of having cognitive problem were included in the clinical PET study. Before PET study, diagnosis of dementia was performed by Japanese-board certified physicians of dementia diagnosis (HH and YN) in a comprehensive diagnosis using clinical diagnostic guideline of Japanese Society of Neurology and others. In details, eligibility criteria of amnestic mild cognitive impairment (MCI) in the present study were based on Petersen’s criteria [14]. Eligibility criteria of Alzheimer’s Disease (AD) in the present study were based on DSM-IV and V and National Institute of Neurogenic, Communicative Disorders and Stroke, AD and Related Disorders Association (NINCDS-ADRDA) [15, 16]. 27 patients with AD were included in the present study (early AD: *n* = 19 and moderate stage AD: *n* = 8) (early AD was defined as AD with MMSE = 20 or more, moderate AD was defined as AD with MMSE = 19 or less). The other 28 were non-AD patients (Supplement in details). All the patients underwent ^18^F-FPYBF-2 PET/CT study. Each patient gave written informed consent. MMSE was performed in each patient within 1 month before PET/CT study. The tracer study for patients was approved by our institutional review boards, the Human Study Committee (approved on Sep. 25, 2013).

**Table 2:** Characteristics of total 55 patients with ad and other dementia or related diseases

Gender, *n* (%)		Age (years)	Age range	MMSE
Male	*n* = 29 (53%)	72.8 ± 9.6	41–88	22.3 ± 4.2
Female	*n* = 26 (47%)	76.1 ± 9.2	48–88	22.7 ± 4.4
Total	*n* = 55	74.4 ± 9.4	41–88	22.5 ± 4.3

Final Diagnosis		Age (years)	MMSE	
			MMSE range	Average score
Early stage AD	*n* = 19	76.0 ± 7.3	20–26	22.5 ± 2.7
Moderate stage AD	*n* = 8	71.1 ± 6.3	11–20	16.5 ± 3.3
Other dementia	*n* = 9	74.9 ± 10.3	13–26	20.2 ± 3.7
Mild cognitive impairment	*n* = 16	77.1 ± 6.1	23–28	25.5 ± 1.6
Normal (cognitively)	*n* = 3	57.0 ± 21.9	28–30	29.3 ± 1.1

SUVR (Mean Cortical Index)	50–60 min (1)	60–70 min (2)	% increase (1) to (2)	50–70 min (average)
Early stage AD	1.275 ± 0.132	1.301 ± 0.136	2.0	1.288 ± 0.134
Moderate stage AD	1.326 ± 0.184	1.358 ± 0.197	2.4	1.342 ± 0.191
Other dementia	1.010 ± 0.054	1.025 ± 0.061	1.5	1.018 ± 0.057
Mild cognitive impairment	1.133 ± 0.136	1.154 ± 0.132	1.9	1.143 ± 0.133
Normal (cognitively)	1.012 ± 0.031	1.022 ± 0.036	1.0	1.017 ± 0.033
Total	1.183 ± 0.171	1.206 ± 0.177	1.9	1.195 ± 0.174

*MMSE* mini mental state examination, *SUVR* standardized uptake value ratio

### Subjects of dual tracer study

From Jan. 2016 to Dec. 2016, 16 subjects (healthy volunteer: 5, patients: 11) (male: 10, female: 6; mean age: 66.3 ± 14.2) (Table 3) were recruited independently for dual tracer study and underwent both ^18^F-FPYBF-2 PET/CT study and ^11^C-PiB PET/CT study on separate days, respectively. Each patient gave written informed consent. MMSE was performed in each patient within 1 month before PET/CT study. This dual tracer study for volunteers and patients was approved by our institutional review boards, the Human Study Committee (approved on Jan. 18, 2016).

**Table 3:** Characteristics of 16 volunteers and patients examined both by PiB and by FPFBF-2

Test subject	Age	Sex	Clinical diagnosis	MMSE score	PiB		FPYBF-2	
						SUVR		SUVR
#1	39	M	Healthy volunteer	25	WNL	0.961	WNL	0.895
#2	41	M	Healthy volunteer^a^	30	WNL	1.045	WNL	1.001
#3	51	M	Healthy volunteer	30	WNL	1.214	WNL	1.087
#4	54	M	Healthy volunteer	30	WNL	1.100	WNL	1.064
#5	70	F	Healthy volunteer	28	WNL	1.079	WNL	1.049
#6	72	M	MCI	24	High	2.285	High	1.374
#7	76	M	MCI	25	High	1.757	High	1.249
#8	82	M	MCI	24	WNL	1.057	WNL	0.862
#9	56	M	Early AD	26	High	1.831	High	1.326
#10	73	F	Early AD	21	High	2.090	High	1.502
#11	76	M	Early AD^c^	19	High	1.756	High	1.322
#12	78	F	Early AD	24	WNL	1.253	High	1.246
#13	78	F	Moderate AD^b^	15	High	2.230	High	1.568
#14	60	F	Other dementia (amyloid angiopathy)	30	WNL	0.932	WNL	0.892
#15	73	F	Other dementia (FTD)	19	WNL	1.238	WNL	1.060
#16	82	M	Other dementia (unknown)	23	WNL	1.134	WNL	0.989
Total	66.3 ± 14.2		24.2 + 44.4		1.435 ± 0.474		1.155 ± 0.219	

*MCI* mild cognitive insufficiency, *MMSE* mini mental state examination, *AD* Alzheimer’s disease, *SUVR* standardized uptake value ratio, *FTD* fronto temporal dementia, *WNL* within normal limit

^a^ Case B, ^b^ Case C, ^c^ Case D in Fig. 6

### PET studies

#### Automated radiosynthesis of [^18^F]FPYBF-2 and [^11^C]PiB

^18^F-FPYBF-2 was prepared in-house. The ^18^F-fluoride was produced with a cyclotron, CYPRIS HM18 [Sumitomo Heavy Industries (SHI), Ltd., Japan] by the ^18^O(p, n)^18^F reaction on 98% enriched ^18^O water. The radiosynthesis of ^18^F-FPYBF-2 was performed using a modification of the methods described by Ono et al. [13] and on a hybrid synthesizer, cassette-type multipurpose automatic synthesizer module (JFE Engineering Corporation, Japan).

^11^C-Pittsburg compound B (^11^C-PiB) was also prepared in-house. The ^11^C-CO_2_ was produced with a cyclotron, CYPRIS HM18 [Sumitomo Heavy Industries (SHI), Ltd., Japan] by the ^14^N(p, α)^11^C reaction on nitrogen gas (0.2% O_2_). The radiosynthesis of ^11^C-PiB was performed using a modification of the methods described by Verdurand et al. [17] and on a hybrid synthesizer, cassette-type multipurpose automatic synthesizer module (JFE Engineering Corporation, Japan).

#### PET data acquisition

In this first clinical volunteer study for newly developed ^18^F-FPYBF-2, 61 cognitively healthy volunteers underwent ^18^F-FPYBF-2 PET study (*n* = 28) or ^18^F-FPYBF-2 PET/CT (*n* = 33), respectively. PET scans were performed by a whole-body PET scanner, GE Advance (pixel size: 2 mm) (GE Healthcare, Waukesha WI, USA), while PET/CT scans were performed by a whole-body PET/CT scanner, Siemens True Point Biograph 16 (pixel size: 1.34 mm) (Siemens/CTI, Erlangen, Germany). Static head PET image acquisition for 20-minutes was performed 50–70 min after the intravenous injection of ^18^F-FPYBF-2 (200 ± 22 MBq). This 20-minute static scan was separately evaluated in two time zones (50–60 min and 60–70 min) for the evaluation of time interval difference in all cases. For the further evaluation of time-activity-curve (TAC) for the brain accumulation of ^18^F-FPYBF-2, 50-minute dynamic PET/CT scan was also performed in 13 volunteers at 0–50 min at the same instant.

In the present clinical patient study using ^18^F-FPYBF-2 for 55 patients with suspected of dementia, ^18^F-FPYBF-2 PET/CT were performed in all subjects. Static head PET image acquisition for 20-minutes was performed 50–70 min after the intravenous injection of ^18^F-FPYBF-2 (204 ± 16 MBq) by a whole-body PET/CT scanner, Siemens True Point Biograph 16 (Siemens/CTI, Erlangen, Germany). This 20-minute static scan was also separately evaluated in two time-zones (50–60 min and 60–70 min) for the evaluation of time interval difference in all cases. For the further evaluation of time-activity-curve (TAC) for the brain accumulation of ^18^F-FPYBF-2, 50-minute dynamic PET/CT scan was also performed in 14 patients with dementia at 0–50 min at the same instant.

In dual tracer study using ^18^F-FPYBF-2 and ^11^C-PiB for 5 volunteers and 11 patients with dementia or related disease, ^18^F-FPYBF-2 PET/CT and ^11^C-PiB PET/CT were performed in all subjects on separate days, independently. Static head PET image acquisition for 20-minutes was performed 50 min after the intravenous injection of ^18^F-FPYBF-2 (213 ± 33 MBq) and ^11^C-PiB (528 ± 57 MBq), respectively. The intervals between these two PET/CT studies were within 2 weeks for patients with dementia and half year for healthy volunteers. PET/CT scans were performed by a whole-body PET/CT scanner, Siemens True Point Biograph 16 (Siemens/CTI, Erlangen, Germany).

For the image data processing in both scanners (the PET scanner and the PET/CT scanner), further information was shown in Supplement.

#### ^18^C-PiB and ^18^F-FPYBF-2 PET template construction

In-house PET template construction was performed for ^11^C-PiB PET and ^18^F-FPYBF-2 PET [18, 19]. Further information was shown in Supplement.

### Automated region of interest analysis

Since the cerebellar cortex can be used as a reference brain region lacking amyloid plaque [6, 20], the Standardized Uptake Value Ratio (SUVR) of each region, indicating amyloid deposition, was calculated as follows;

$${\text{SUVR}}={\text{SUV}}\;{\text{brain}}/{\text{SUV}}\;{\text{cerebellar}}\;{\text{cortex,}}$$

where SUV brain and SUV cerebellar cortex indicate SUV in each brain region and the cerebellar cortex, respectively. To obtain quantitative regional SUVR values of ^18^F-FPYBF-2 PET and ^11^C-PiB PET, we performed automated region of interest (ROI) analyses. The Automated Anatomical Labeling atlas (AAL) [21], which is publicly and widely available from the Internet, e.g. in open source software packages (MRIcro/MRIcron, http://www.mricro.com/), were used as template-based predefined ROIs. The AAL atlas consists of 45 anatomical ROIs in each hemisphere and a cerebellar parcellation with 26 ROIs [22]. The AAL ROIs were finally masked with the gray matter defined by the MNI152 standard-space T1-weighted average structural template image available from the FSL software (http://www.fmrib.ox.ac.uk/fsl) and used as the predefined ROIs because the original AAL ROIs tend to be large and extend to the margin of the gray matter.

The reconstructed ^18^F-FPYBF-2 PET and ^11^C-PiB PET images were spatially normalized to a standard MNI space by the DCT-based approach [19] implemented in SPM8 with the in-house ^18^F-FPYBF-2 PET and ^11^C-PiB PET templates, respectively. The spatial normalization of amyloid PET images by the amyloid PET template was in accordance with the previous procedure for ^11^C-PIB [23]. We also confirmed visually that inversely transformed AAL ROIs to an individual space corresponded to PET images in each subject. All AAL ROIs in the standard MNI space were inversely transformed to individual spaces by SPM8 using the inverse deformation field. Since these individual ROIs are automatically defined, the operator induced bias in defining ROIs manually can be avoided [24]. The cerebellar parcellation with 26 ROIs were combined and used as a reference region to create SUVR images. Mean SUVR values within 90 anatomical ROIs in both hemispheres were calculated by an in-house Matlab script.

Finally, as a representative value for cortical amyloid plaque deposition of each subject, the Mean Cortical Index was defined as a mean SUVR value within the frontal, posterior cingulate, precuneus, parietal and lateral temporal cortical regions [25].

### Statistics

All values are expressed as mean ± SD. All the statistical analysis was performed using statistical software, JMP 8J version (SAS Institute, Cary NC, USA), in which *p* values < 0.05 were considered statistically significant. A comparison between each group was analyzed by the Wilcoxon or the Kruskal–Wallis Analysis for unpaired data. Correlation coefficient analysis between SUVR values of ^18^F-FPYBF-2 and ^11^C-PiB was performed by Pearson’s analysis.

## Results

### Volunteers

First clinical PET studies for volunteers were performed in 61 healthy volunteers. MMSE test showed high score in all the age range between 24 and 79 years old (Table 1). In this volunteer study, three subjects out of 64 volunteers were excluded. Further information was shown in Supplement.

**Fig. 1:**
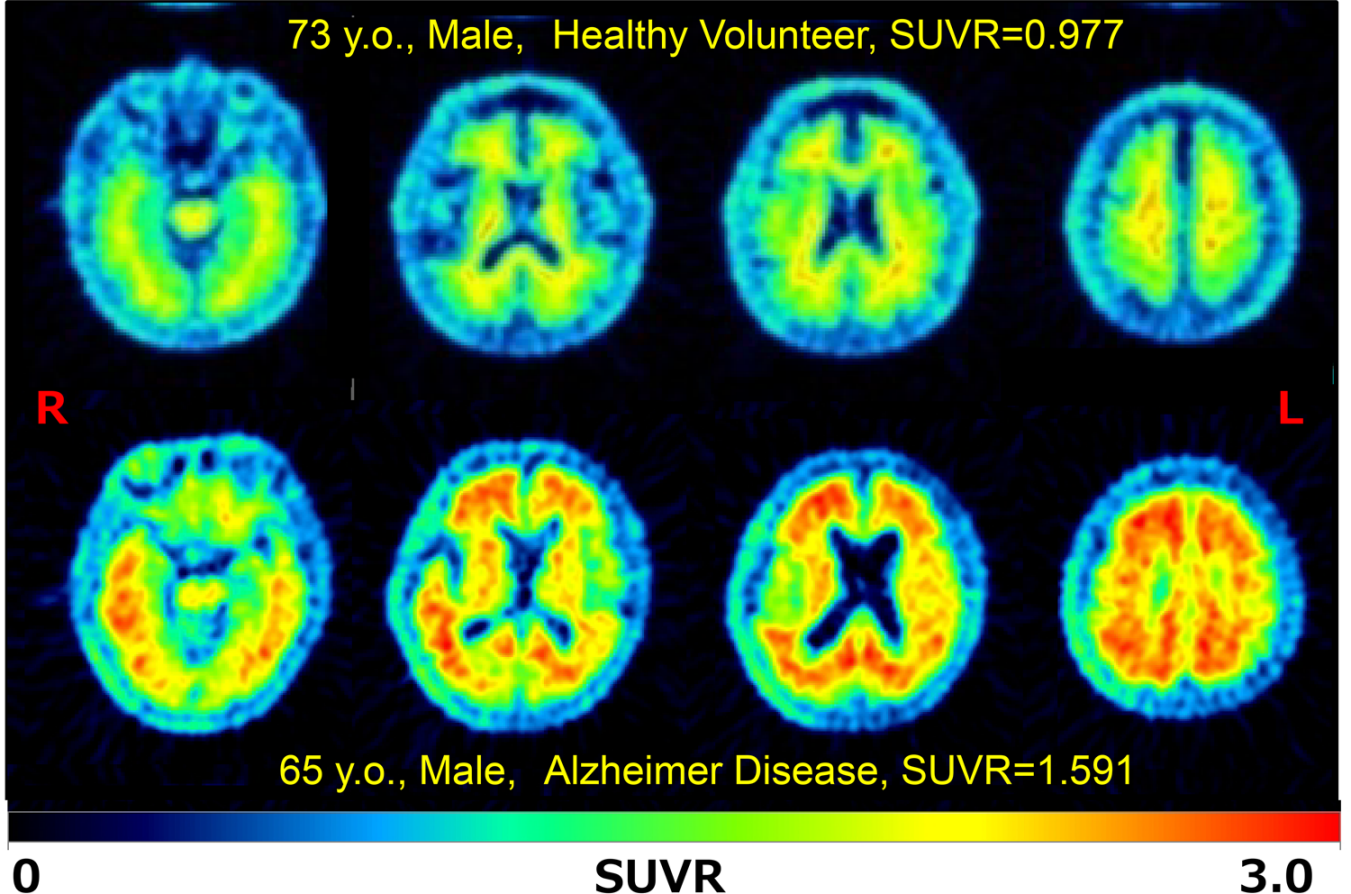
Representative brain axial PET images of ^18^F-FPYBF-2 PET in a healthy volunteer and a patient with Alzheimer disease. The upper half showed a typical case of healthy 73 years old male volunteer, who has MMSE: 28 with Mean Cortical Index of SUVR as 0.977. The lower half showed a typical case of patient with Alzheimer disease, 65 years old male, who has MMSE: 14 with Mean Cortical Index of SUVR as 1.591. Prominent accumulation of ^18^F-FPYBF-2 was observed in gray matter of frontotemporal cortex, parietal and occipital cortex and posterior cingulate gyrus

The present volunteer study showed that ^18^F-FPYBF-2 uptake was mainly observed in cerebral white matter (Fig. 1) and that average Mean Cortical Index at 50–70 min after injection was low and stable (1.066 ± 0.069). Average Mean Cortical Index of cognitively healthy volunteers calculated both at PET study (*n* = 28, 1.049 ± 0.057) and at PET/CT (*n* = 33, 1.064 ± 0.074) showed no significant difference. Average Mean Cortical Index of healthy men (age: 55.6 ± 13.6) at 50–70 min after injection (1.044 ± 0.064) was slightly lower than those of healthy women (age: 53.4 ± 13.5) (1.076 ± 0.067), but the difference was not significant (*p* = 0.57) (Table 1).

**Fig. 2:**
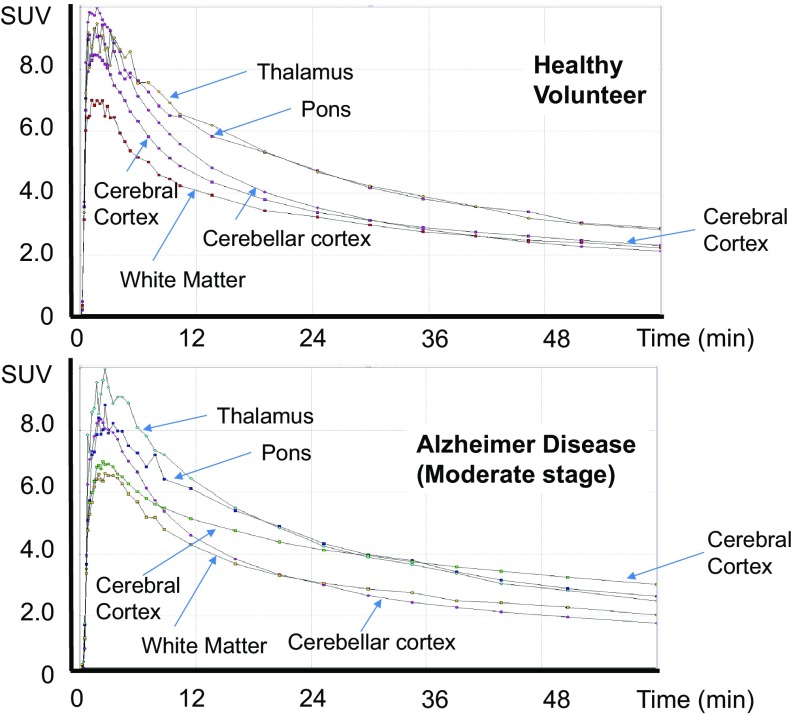
Time-activity-curves (TAC) of representative two cases; healthy volunteer (upper) and Alzheimer disease (moderate stage) (bottom). Regional TACs were shown for cerebral cortex (including frontotemporal lobe), white matter, cerebellar cortex, thalamus and pons. Subjects who showed similar TACs to mean TACs of each group were selected. Radioactivity was shown as Standardized Uptake Value (SUV) in this figure. In AD patients, TAC of cortex showed higher retention than that in healthy volunteers, and reached a plateau similar to the level of thalamus and pons

In the evaluation of 50-min dynamic PET/CT scan, time-activity-curve (TAC) for the cortical accumulation was calculated in 13 volunteers at the same instant and the SUV of cortex showed equilibrium phase with the similar level of that of cerebellar cortex at 50–70 min after injection of ^18^F-FPYBF-2 (Fig. 2).

### Patients

The 55 patients including 27 AD patients who were suspected of having dementia underwent ^18^F-FPYBF-2 PET/CT in clinical PET/CT study. MMSE test showed relatively higher score in MCI patients, while it showed lower score in AD and other dementia patients (Table 2). No adverse events were reported during at least 1-year follow-up period.

**Fig. 3:**
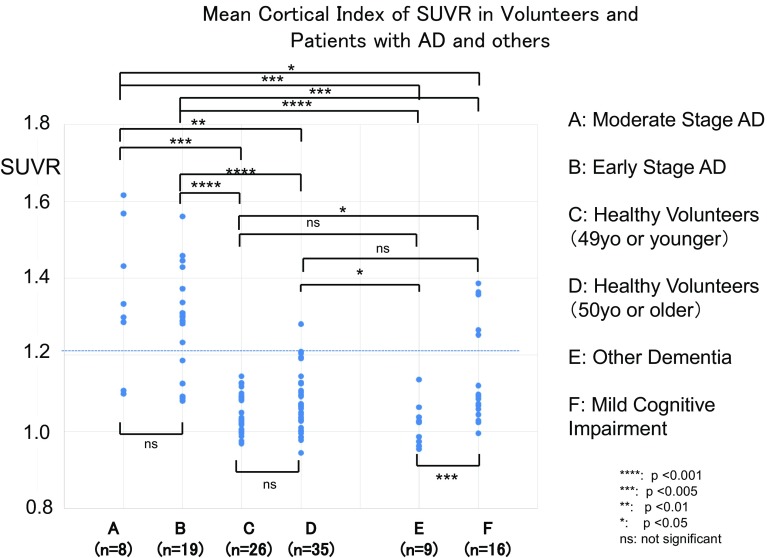
Results of Mean Cortical Index of SUVR in patients suspected of having dementia and related diseases. Each group represents a patient group with various disease, A: AD in moderate stage, B: AD in early stage, C: Healthy volunteers (49 years old and younger), D: Healthy volunteers (50 years old and older), E: Other dementia (including Dementia with DLB, PART, FTD, iNPH, corticodegeneration and unknown), and E: Mild Cognitive impairment (MCI). Mean Cortical Index of AD patients in moderate stage (1.342 ± 0.191) and early stage (1.288 ± 0.134) were significantly higher than those of healthy volunteers in both age range (50 years old and older and 49 years old and younger). Based on the regression line in Fig. 4a (dotted line), we determined that Mean Cortical Index of 1.2 in [18F]FPYBF-2, which corresponds to the widely accepted threshold of 1.5 for PiB, can be used as a threshold value between normal and dementia. Please find Fig. 4

Clinical studies with 27 AD patients showed that ^18^F-FPYBF-2 uptake was observed in cerebral gray matter as well as cerebral white matter (Fig. 1). In the evaluation of 50-minute dynamic PET/CT scan, TAC for the cortical accumulation of AD patients showed higher retention and reached higher equilibrium level than that of cerebellar cortex (Fig. 2). Average Mean Cortical Index at 50–70 min after injection were high in AD patients with moderate (1.342 ± 0.191) and early stage (1.288 ± 0.134), which were significantly higher than those of healthy volunteers (49 years old or younger and 50 years old or older) (*p* < 0.01, *p* < 0.001) (Fig. 3). There was no significant difference between the Mean Cortical Index of early stage AD and moderate stage AD patients.

**Fig. 4:**
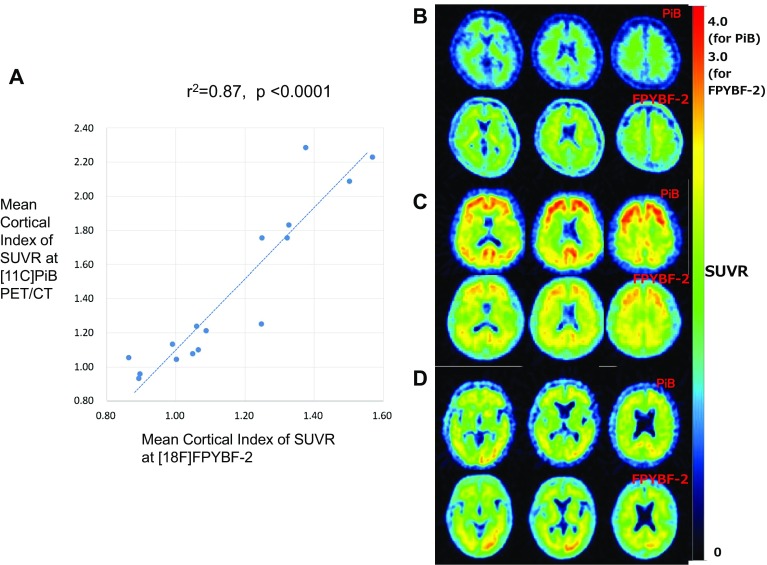
Comparison of Mean Cortical Index of SUVR at ^11^C-PiB and ^18^F-FPYBF-2 in the dual tracer study (**a**). Excellent linear correlation was observed between those of ^11^C-PiB and ^18^F-FPYBF-2 with statistical values (*r*^2^ = 0.87, *p* < 0.0001). Comparison of axial brain images of ^11^C-PiB (top three images in each case) and ^18^F-FPYBF-2 (bottom in each case) in three cases for example: **a** a case of normal volunteer (30 years old, male, MMSE = 30), **b** a case of moderate stage AD (78 years old, female, MMSE = 15), and **c** a case of early stage AD with a history of traumatic subarachnoid hemorrhage (76 years old, male, MMSE = 19). In the third case, amyloid deposition in left occipital lobe was prominent compared to right lobe both at ^11^C-PiB and ^18^F-FPYBF-2. Please note that window level of SUVR was set 0–3.0 for ^18^F-FPYBF-2 because of its narrow dynamic range, while it was set 0–4.0 for ^11^C-PiB

### Subjects of dual tracer study

Sixteen subjects underwent both ^18^F-FPYBF-2 PET/CT study and ^11^C-PiB PET/CT study on separate days, respectively (Table 3). Final diagnosis of these 16 subjects were as follows; healthy volunteer: *n* = 5, MCI: *n* = 3, early stage AD: *n* = 4, moderate stage AD: *n* = 1, other dementia (amyloid angiopathy, FTD and unknown): *n* = 3. In this table, the diagnostic classification (high or WNL) was performed based on the quantitative analysis of Mean Cortical Index. Average Mean Cortical Index at 50–70 min after injection were higher at ^11^C-PiB PET/CT study (1.435 ± 0.474), while its standard deviation was wider. On the other hand, average SUVR at ^18^F-FPYBF-2 PET/CT study was lower and stable with its smaller standard deviation (1.155 ± 0.219). Mean Cortical Index of SUVR at both studies showed an excellent linear correlation between each other (correlation coefficient, *r*^2^ = 0.87, *p* < 0.0001) (Fig. 4 left). The results in Fig. 4 left indicate that 18F-FPYBF-2 has lower lesion-to-normal contrast than 11C-PiB both in SUVR and in images. Regression line in Fig. 4 left showed that Mean Cortical Index of 1.2 in ^18^F-FPYBF-2 corresponded to Mean Cortical Index of 1.5 in ^11^C-PiB. Uptake pattern of ^18^F-FPYBF-2 and ^11^C-PiB in each brain region was also similar with each other. Using this threshold value for ^18^F-FPYBF-2, Mean Cortical Index of 1.2, the average SUVR for amyloid positive cases in AD (*n* = 20) and healthy volunteers excluding amyloid positive cases in volunteer study (*n* = 57) were 1.372 ± 0.110 and 1.054 ± 0.055 (mean ± SD), respectively. Figure 4 right showed brain images of three cases for example: a case of normal volunteer, a case of moderate stage AD, and a case of early stage AD with a history of traumatic subarachnoid hemorrhage. In the third case, amyloid deposition in left occipital lobe was prominent both at ^18^F-FPYBF-2 PET/CT study and ^11^C-PiB PET/CT study.

## Discussion

Our first clinical study clearly indicated that ^18^F-FPYBF-2 is a safe and stable amyloid PET tracer with longer half-life with F-18 and is comparable to ^11^C-PiB in the detectability of amyloid deposition with high linear correlation, and that ^18^F-FPYBF-2 PET/CT is a useful and reliable diagnostic tool for the evaluation of AD. Although ^18^F-FPYBF-2 is a “late” amyloid PET tracer after the appearance of several tracers in clinical practice with comparable diagnostic ability, we would like to show the potential of ^18^F-FPYBF-2 as diagnostic abilities as an amyloid imaging tracer and expand the utilization of this tracer further in various fields of research and clinical practice in the following sentences.

The diagnostic abilities of ^18^F-FPYBF-2 as an amyloid imaging tracer are satisfactory and comparable to the other amyloid PET tracers (Figs. 1, 3). Figure 3 showed that differential diagnosis between AD patients and healthy volunteers was achieved using the qualitative analysis of Mean Cortical Index of SUVR, and that the threshold of Mean Cortical Index was about 1.2. Figure 4 left also showed that Mean Cortical Index of 1.2 in ^18^F-FPYBF-2 closely corresponded to Mean Cortical Index of 1.5 in ^11^C-PiB. It is known that Mean Cortical Index of 1.5 has been used as a threshold between AD patients and healthy volunteers in ^11^C-PiB PET/CT study [26, 27]. Although the threshold value is different, we believe that diagnostic performance of ^18^F-FPYBF-2 PET/CT using quantitative analysis of Mean Cortical Index is enough in the differential diagnosis of AD from healthy volunteers. We have to admit that the threshold value of Mean Cortical Index 1.2 could not clearly separate AD and healthy volunteers. In the present study, there were several AD patients with low amyloid deposition and several persons with high amyloid deposition in healthy volunteer group. However, we believe that this is reasonable in clinical study. As we mentioned above, our clinical diagnosis of AD was not confirmed with pathology or others. In addition, it is known that about 10–30% of healthy aged persons showed high amyloid deposition [28]. Therefore, we may say that our data of amyloid positive rate are comparable with the previously published data with other amyloid tracers.

While typical cases shown in Fig. 1 can be clearly diagnosed visually, we were under impression that visual diagnosis would not be easy in ^18^F-FPYBF-2 PET/CT. As shown in Fig. 4 and Table 3, Mean Cortical Index of AD patients observed at ^18^F-FPYBF-2 PET/CT is relatively lower than that at ^11^C-PiB PET/CT, which means that ^18^F-FPYBF-2 has a narrower dynamic range and lower lesion-to-normal contrast than ^11^C-PiB both in SUVR and in images. It may be said that the impact of ^18^F-FPYBF-2 as a PET imaging tool of AD is not so attractive in a visual sense compared to that of ^11^C-PiB.

Several amyloid PET tracers have been developed and comparative study between ^11^C-PiB and each PET tracer was performed so far [29–33]. While some of the tracers also showed the narrower dynamic range than that of ^11^C-PiB, most of these reports revealed that diagnostic abilities of these amyloid PET tracers were similar and identical to that of ^11^C-PiB. In these PET tracers, its own specific method for visual and quantitative diagnosis is proposed in each tracer, including the indication of color or black and white tones for image display, the indication of analyzed area of brain, etc. We have to admit that we could not establish the most appropriate specific visual diagnostic method for ^18^F-FPYBF-2 PET/CT, so far. For the establishment of visual diagnosis, further evaluation of appropriate color scaling with comprehensive interpretation or new diagnostic method with regional area analysis or others would be needed.

It is particularly worth noting that evaluation of brain amyloid deposition in healthy volunteers was performed in broad spectrum of age range from 24 to 79 in age. Our data showed that average Mean Cortical Index of healthy volunteers (20–39, 40–49, 50–59, 60–69, 70–79 years old) were almost similar, except for the difference between the group 20–39 years old and the group 70–79 years old (*p* < 0.05) (Supplement Fig. 1). A slight upward extension of the distribution was due to appearance of high SUVR subjects in the older age range. This is reasonable because it is known that, as the age increases, some normal subjects present amyloid deposition while others remain amyloid negative. Our data of 8 healthy young volunteers in the age of 20–39 can be used as a control group for the evaluation of premature senility, which is often observed in patients with Down syndrome. It is known that adults with Down syndrome even at its younger age are at a very high risk of developing early onset AD due to trisomy of chromosome 21 [34]. We are planning to have further research using ^18^F-FPYBF-2 PET/CT as a diagnostic tool for the evaluation of early onset AD in Down syndrome in near future.

The limitation of the present study should be addressed. First of all, three-dimensional magnetic resonance (MR) images were not available in the study. Because the predefined AAL ROIs were masked with the gray matter of averaged standard T1-weighted structural template image in all subjects, the SUVR values in the study were influenced by inter-subject variability of gray matter volumes and the partial volume effect. Furthermore, the spatial normalization in the study was based on amyloid PET images by the amyloid PET template. Although we visually confirmed that inversely transformed AAL ROIs to an individual space corresponded to PET images in each subject, the accuracy of spatial normalization might be lower compared with MR-based normalization. The more sophisticated methods of spatial normalization only using PET images would improve both the accuracy of spatial normalization and quantification [35–37]. Second, the clinical diagnosis of AD patients and other related disease was performed before PET study by board certified physicians in a comprehensive diagnosis using Japanese clinical diagnostic guideline. Therefore, there was no confirmation of the diagnosis by pathological examination or autopsy, or biomarkers in cerebrospinal fluids, such as Aβ40, Aβ42 and phosphorylated Tau (pTau). Third, in the present study, 16 patients of Mild Cognitive Impairment (MCI) showed inconclusive results of Mean Cortical Index. The results of Mean Cortical Index of SUVR in MCI patients showed relatively high and could not be clearly distinguished with those of AD patients. However, we believe that this result was reasonable. MCI was just a diagnosis at the time before PET study. It is possible that within these MCI patients there must be several patients included who will develop AD in future. Further follow-up study would be needed to clarify the outcome of the patients with high amyloid deposition.

In conclusions, our first clinical study showed that ^18^F-FPYBF-2 is a safe and stable amyloid PET tracer with longer half-life with F-18 and its diagnostic ability is comparable to ^11^C-PiB. In addition, it can be said that ^18^F-FPYBF-2 PET/CT is a useful and reliable diagnostic tool for the evaluation of AD by the quantitative analysis using Mean Cortical Index of SUVR, which could clearly distinguish Alzheimer disease patients by threshold of 1.2.

## Electronic supplementary material

Below is the link to the electronic supplementary material.

Supplementary material 1 (PPT 880 KB)

Supplementary material 2 (DOCX 31 KB)
