# Supplementary material for: 18F-FPYBF-2, a new F-18-labelled amyloid imaging PET tracer: first experience in 61 volunteers and 55 patients with dementia
Source: Ann Nucl Med. 2018 Jan 31;32(3):206–16. doi: 10.1007/s12149-018-1236-1 (PMC5852179; doi:10.1007/s12149-018-1236-1)
Supplement: Supplementary file 1 — Supplementary material 1 (PPT 880 KB) [file 12149_2018_1236_MOESM1_ESM.ppt]

## Slide 1
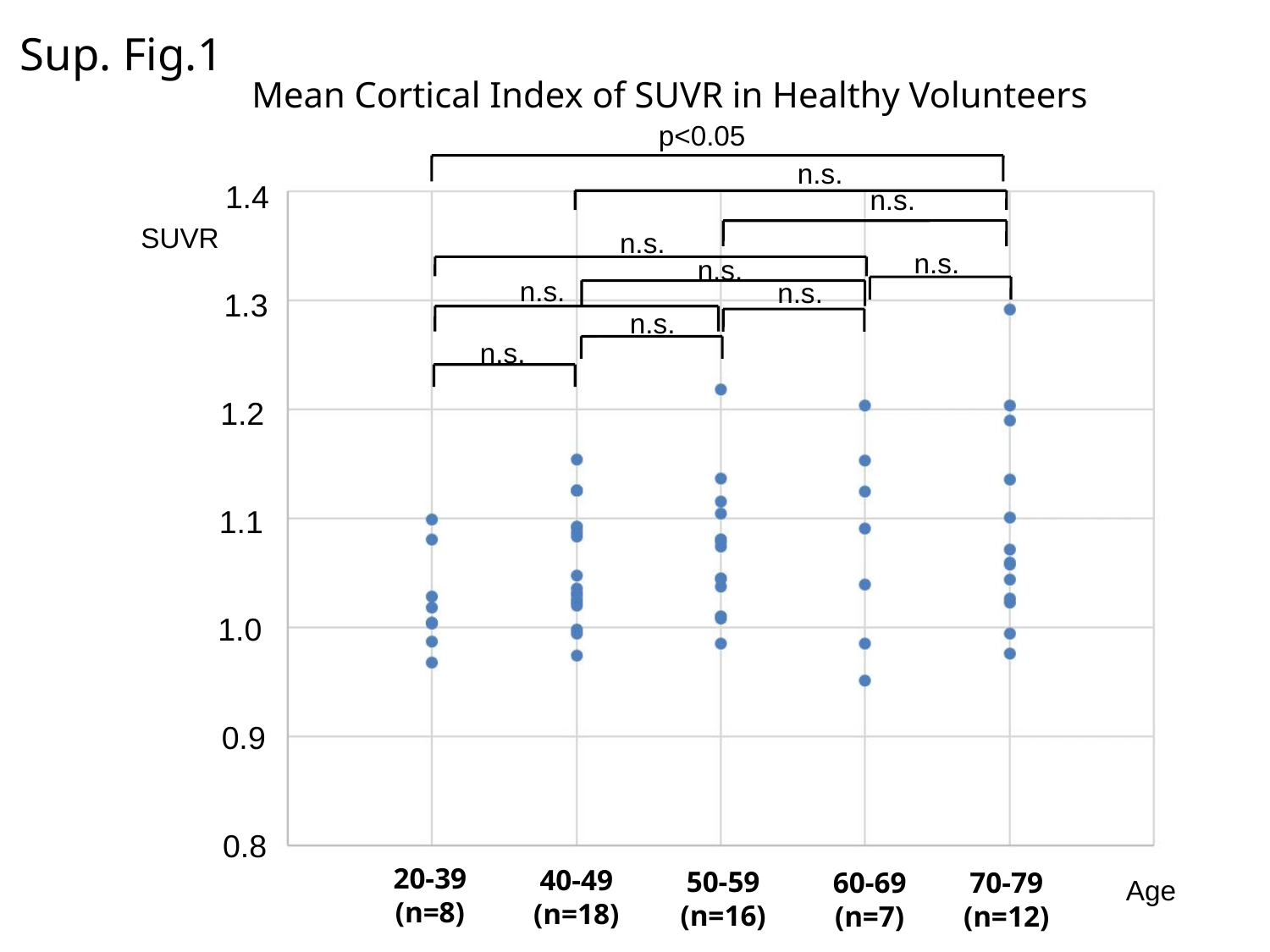

Sup. Fig.1
Mean Cortical Index of SUVR in Healthy Volunteers
p<0.05
n.s.
1.4
n.s.
SUVR
n.s.
n.s.
n.s.
n.s.
n.s.
1.3
n.s.
n.s.
1.2
1.1
1.0
0.9
0.8
20-39
(n=8)
40-49
(n=18)
50-59
(n=16)
60-69
(n=7)
70-79
(n=12)
Age

## Slide 2
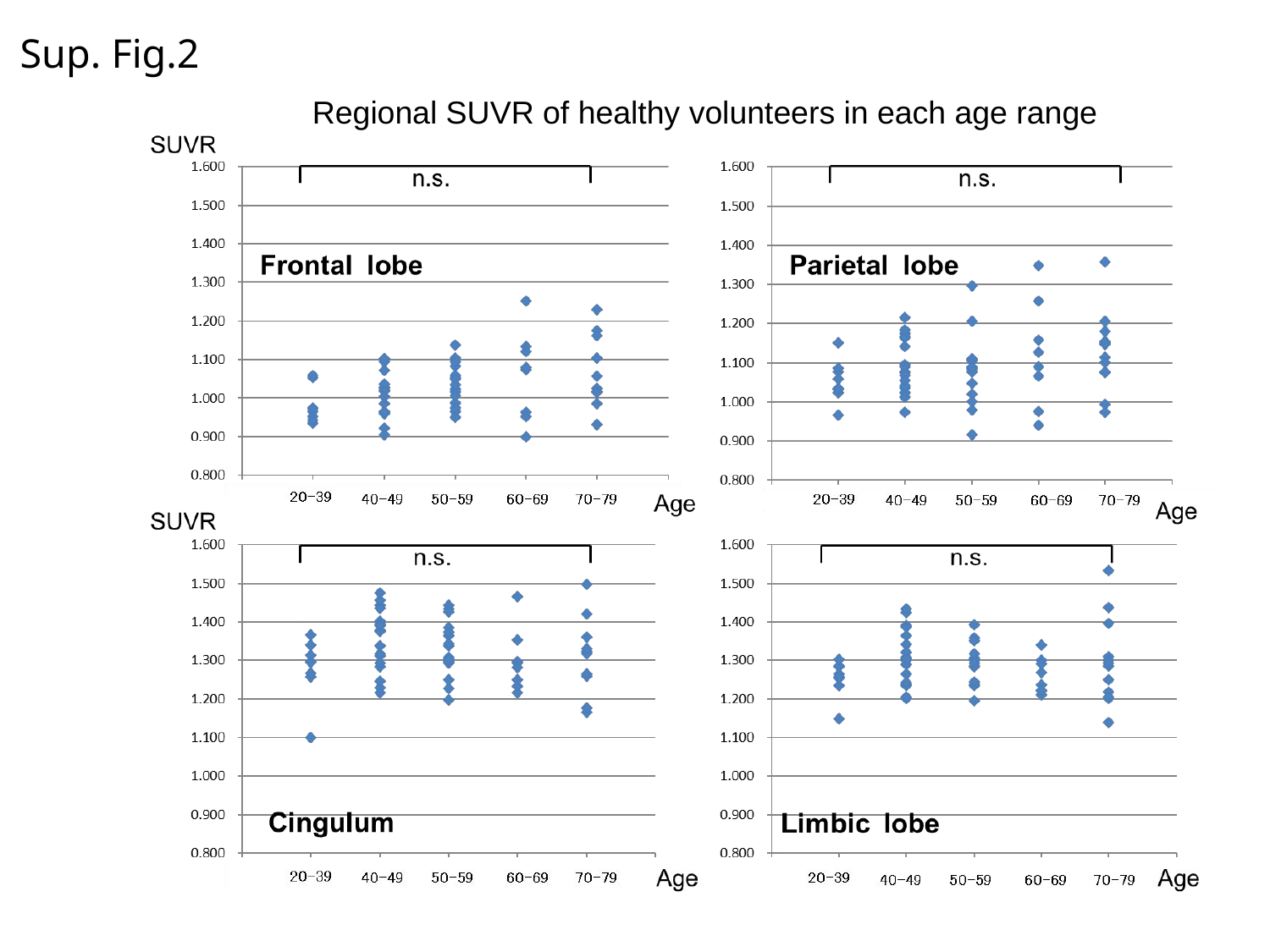

Sup. Fig.2
Regional SUVR of healthy volunteers in each age range

## Slide 3
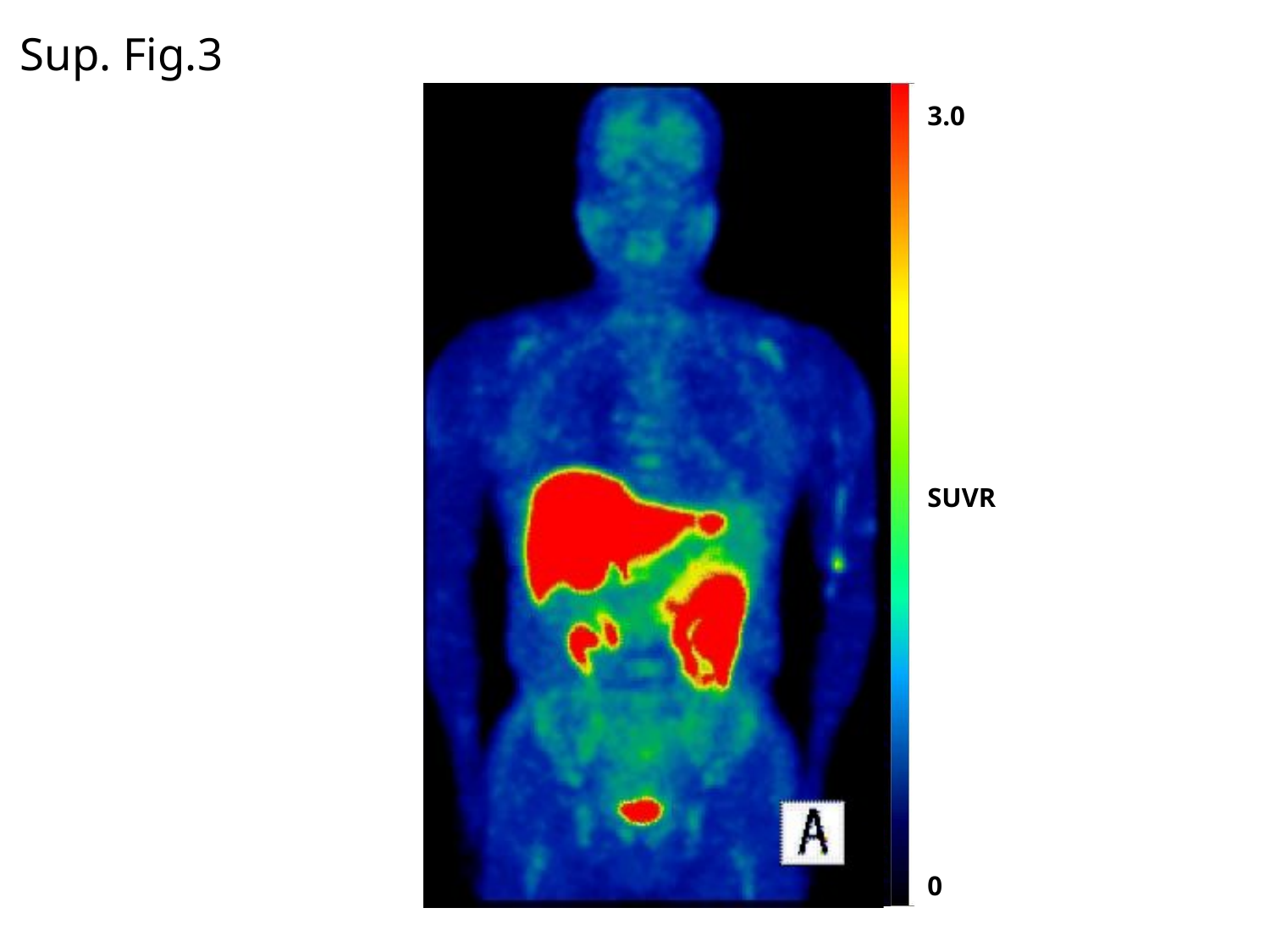

Sup. Fig.3
3.0
SUVR
0

## Slide 4
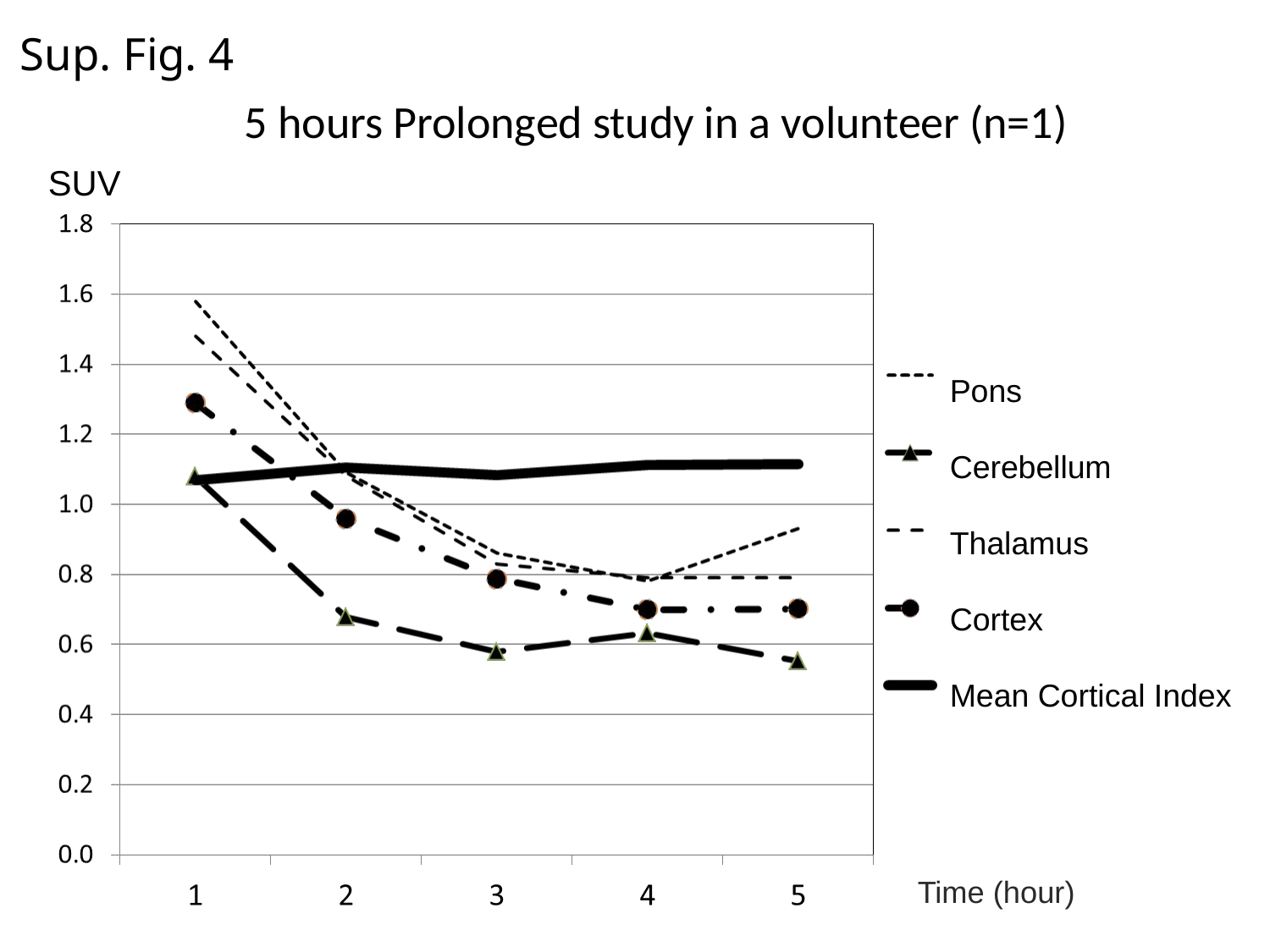

Sup. Fig. 4
5 hours Prolonged study in a volunteer (n=1)
SUV
Pons
Cerebellum
Thalamus
Cortex
Mean Cortical Index
Time (hour)

## Slide 5
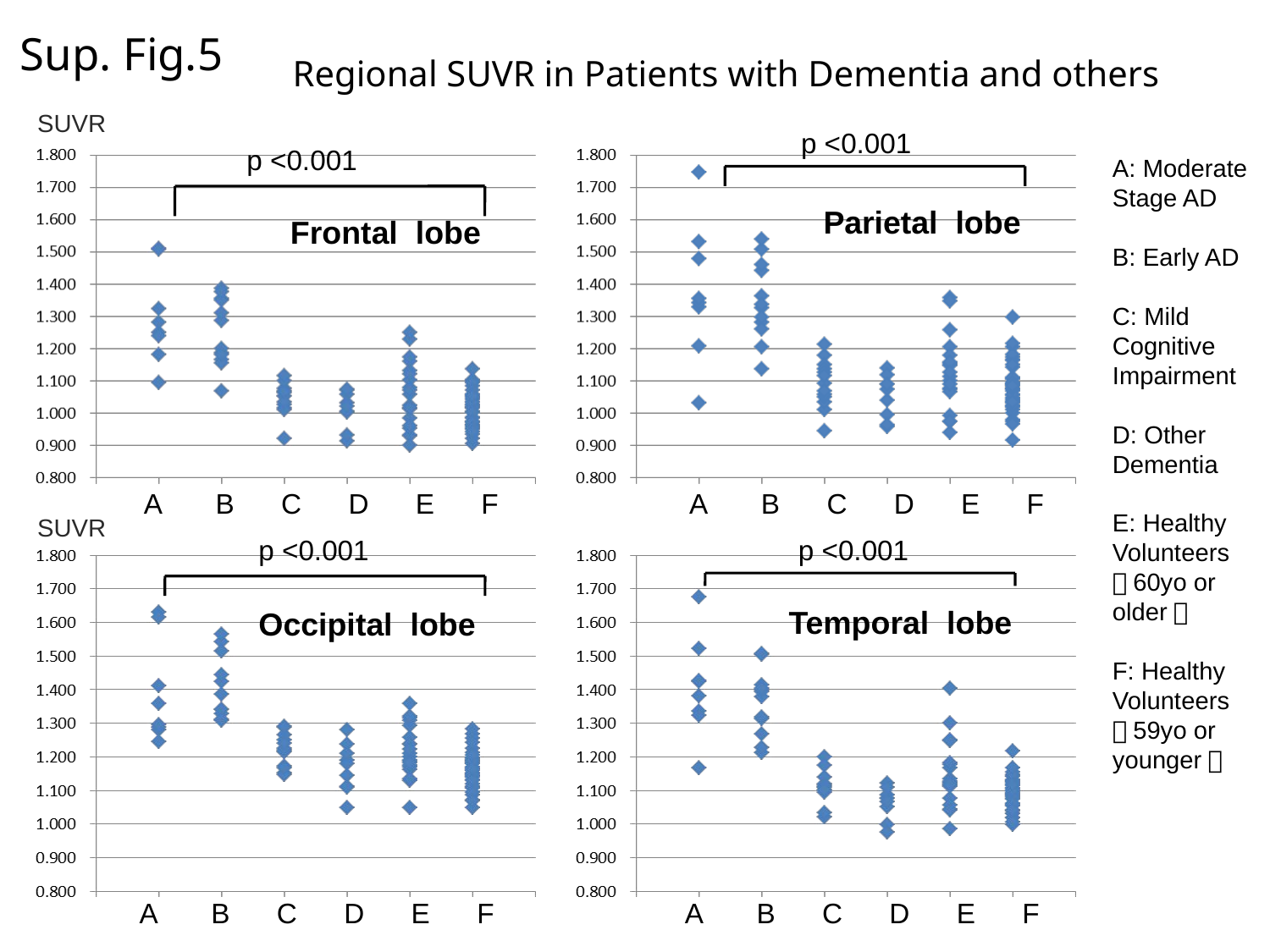

Sup. Fig.5
Regional SUVR in Patients with Dementia and others
SUVR
p <0.001
p <0.001
A: Moderate Stage AD
B: Early AD
C: Mild Cognitive Impairment
D: Other Dementia
E: Healthy Volunteers（60yo or older）
F: Healthy Volunteers（59yo or younger）
Parietal lobe
Frontal lobe
A B C D E F
A B C D E F
SUVR
p <0.001
p <0.001
Temporal lobe
Occipital lobe
A B C D E F
A B C D E F

## Slide 6
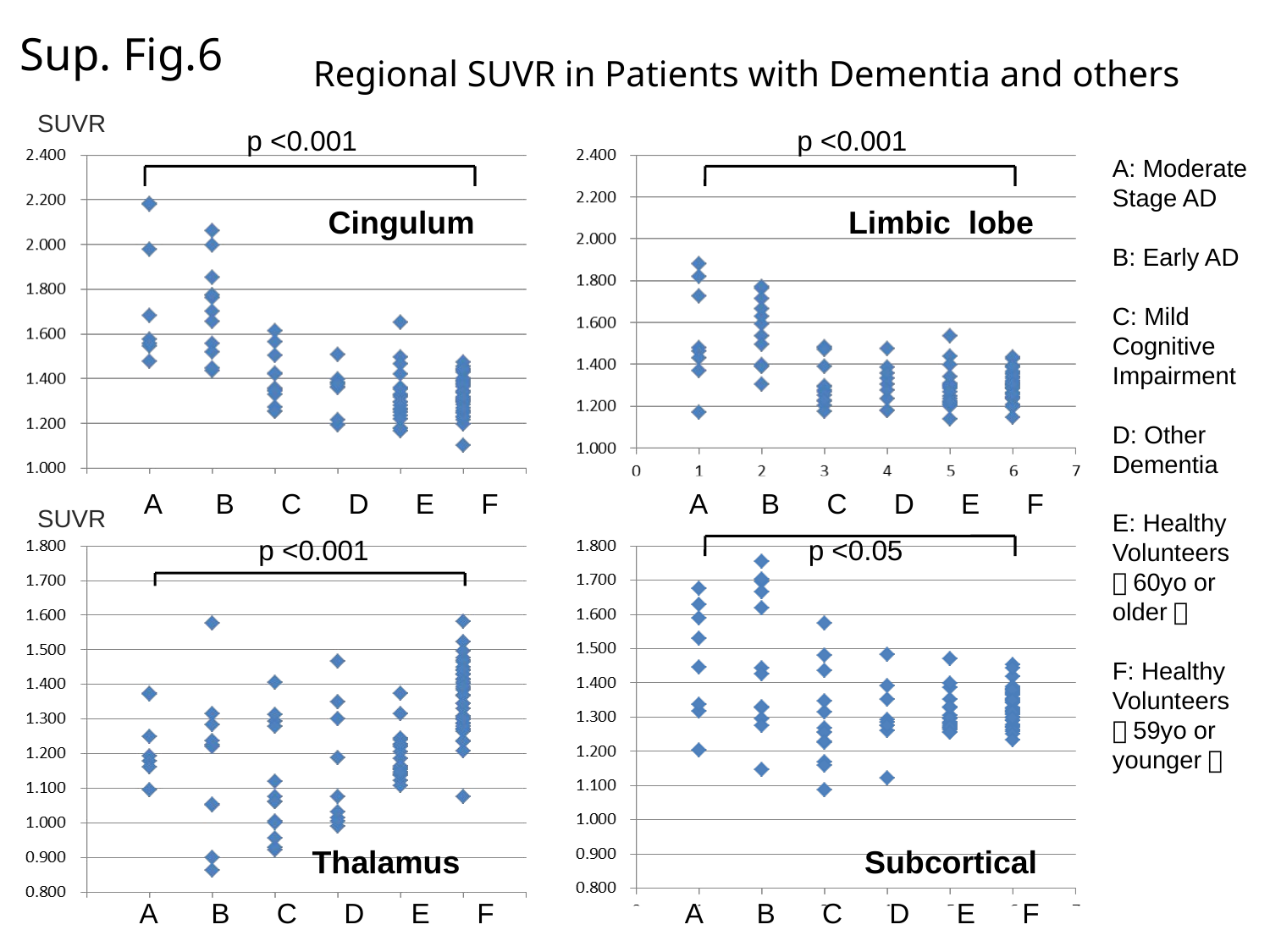

Sup. Fig.6
Regional SUVR in Patients with Dementia and others
SUVR
p <0.001
p <0.001
A: Moderate Stage AD
B: Early AD
C: Mild Cognitive Impairment
D: Other Dementia
E: Healthy Volunteers（60yo or older）
F: Healthy Volunteers（59yo or younger）
Cingulum
Limbic lobe
A B C D E F
A B C D E F
SUVR
p <0.001
p <0.05
Thalamus
Subcortical
A B C D E F
A B C D E F
