# Supplementary material for: 18F-FPYBF-2, a new F-18-labelled amyloid imaging PET tracer: first experience in 61 volunteers and 55 patients with dementia
Source: Ann Nucl Med. 2018 Jan 31;32(3):206–16. doi: 10.1007/s12149-018-1236-1 (PMC5852179; doi:10.1007/s12149-018-1236-1)
Supplement: Supplementary file 2 — Supplementary material 2 (DOCX 31 KB) [file 12149_2018_1236_MOESM2_ESM.docx]

**Supplement Text For**

**^18^F-FPYBF-2, a new F-18 labelled amyloid imaging PET tracer**

- **First experience in 61 volunteers and 55 patients with dementia –**

**MATERIALS AND METHODS**

***Patients***

Twenty-seven patients with AD were included in the present study (early AD: n=19 and moderate stage AD: n=8) (early AD was defined as AD with MMSE=20 or more, moderate AD was defined as AD with MMSE=19 or less). In the present study, patients with AD included “probable AD” only. Probable AD was diagnosed based on NINCDS-ARDRA criteria and DSM-IV. “Possible AD” was not included. Patients with “possible AD” were classified as unknown non-AD patients, mentioned below. The other 28 non-AD patients who also underwent PET study were as follows; other dementia: n=9 (including Dementia with Lewy Bodies (DLB) (n=3), Frontotemporal Dementia (FTD) (n=1), idiopathic Normal Pressure Hydrocephalus (iNPH) (n=1), corticodegeneration (n=1) and unknown (n=3)), Mild Cognitive Impairment (MCI): n=16, cognitively normal patients: n=3.

**Dosimetry and pharmacokinetics of [^18^F]FPYBF-2**

For evaluation of radiation exposure and pharmacokinetics, volunteer studies were performed in four additional volunteers (male: n=3, female: n=1, age range: 25-56), where a 10-minutes dynamic PET/CT scan of the body (chest and abdomen) was performed at 0-10min and a 12-minutes whole-body static scan was performed 5 times during 15-90min after the injection of [^18^F]FPYBF-2. Mean effective whole body dose of radiation exposure was calculated by OLINDA/EXAM ver.2 (Vanderbilt University TENNESSEE, U.S.). Measurement of blood and plasma radioactivity of metabolites was performed at 15 and 60 min after the injection of [^18^F]FPYBF-2.

***PET Data Acquisition***

For the image data processing in both scanners (the PET scanner and the PET/CT scanner), the transaxial effective fields of view of these scanners were 256 and 342 mm in diameter, and the matrix sizes were 128 x 128 and 256 x 256, respectively. All acquisition data were reconstructed using the back projection reconstruction both in PET and PET/CT. In reconstruction of PET data, images were blurred to 8.0mm full width at half maximum in the transaxial direction using a Hanning filter. In PET/CT data, images were blurred to 6.0mm full width at half maximum in the transaxial direction using a Gaussian filter. For the PET/CT scanner, the CT data were used for attenuation correction. Dynamic scan of brain was performed in 14 volunteers. Tissue-activity curve (TAC) of brain accumulation of ^18^F-FPYBF-2 was evaluated using PMOD version 3.204 (PMOD Technologies Ltd., Zurich, Switzerland).

***^18^C-PiB and ^18^F-FPYBF-2 PET Template Construction***

Ten three-dimensional T1-weighted magnetic resonance (MR) and ^11^C-PiB PET images of subjects (6 females and 4 males; age ± SD = 77.7 ± 4.5) with mild cognitive decline were used for an in-house ^11^C-PiB PET template construction. Each ^11^C-PiB PET image during the first 10 min, which well delineates cerebral cortices, was co-registered to each MRI with rigid transformation using Statistical Parametric Mapping (SPM) version 8 (Wellcome Department of Cognitive Neuroscience, London, UK). Each ^11^C-PiB PET image during 50–70 min was also co-registered to each MRI with the same parameter. The MR images were spatially normalized using the diffeomorphic anatomical registration using the exponentiated Lie algebra (DARTEL) algorithm [17] implemented in a VBM8 toolbox (<http://dbm.neuro.uni-jena.de/vbm>) with standard parameters. Then, the co-registered ^11^C-PiB PET images during the first 10 min and 50–70 min were, respectively, spatially normalized to a standard Montreal Neurological Institute (MNI) space by the forward deformation field obtained from each MRI normalization. Finally, template ^11^C-PiB PET images during the first 10 min and 50–70 min were generated by averaging the ten normalized images and smoothed with an 8-mm full-width at half-maximum (FWHM) isotropic Gaussian kernel.

Twenty-four ^18^F-FPYBF-2 PET images obtained by a 60 min dynamic scan of healthy subjects (15 females and 9 males; age ± SD = 57.5 ± 9.5) were used for an in-house ^18^F-FPYBF-2 PET template construction. Each ^18^F-FPYBF-2 PET image during 50–60 min was co-registered to each image during the first 10 min. The PET images during the first 10 min were spatially normalized by the discrete cosine transform (DCT)-based approach [18] implemented in SPM8 with the first 10 min ^11^C-PiB PET template. Then, the co-registered ^18^F-FPYBF-2 PET images during 50–60 min were spatially normalized with the same parameter obtained from the first 10 min image normalization. Finally, a template ^18^F-FPYBF-2 PET image during 50–60 min was generated by averaging the 24 normalized images and smoothed with an 8-mm FWHM isotropic Gaussian kernel.

**Results**

***Automated radiosynthesis of [^18^F]FPYBF-2 and [^11^C]PiB***

The radiochemical purity of the [^18^F]FPYBF-2 was more than 98%. Specific activity of [^18^F]FPYBF-2 was about 339 GBq/µmol (mean). The radiochemical purity of the [^11^C]PiB was more than 99.9%. Specific activity of [^11^C]PiB was 95.5 ± 10.1 GBq/µmol (mean ± SD). Further information in detail will be reported in another paper (in preparation). Preclinical studies including the quality control tests of final products and safety assessment were already performed with successful results and will be reported in another paper (in preparation). The products of [^18^F]FPYBF-2 and [^11^C]PiB meet the relevant quality standard such as the Quality Control of [^18^F]FDG. There was no harmful finding at an extended single intravenous dose toxicity test for Active ingredient of [^18^F]FPYBF-2. There was no harmful finding at a single dose toxicity test for radiolabeled final products of [^18^F]FPYBF-2 and [^11^C]PiB.

***Volunteers***

First clinical volunteer PET studies were performed in 61 healthy volunteers. No adverse events were reported during at least one-year follow-up period. MMSE test showed high score in all the generation between 24-79 years old (Table 1). In this volunteer study, three subjects out of 64 volunteers were excluded. A female of age 70 who showed lower MMSE score (25) and showed mild cognitive impairment were excluded. A female of age 64 who showed normal MMSE score (29) but was diagnosed as mild cognitive impairment by our neurological clinic were excluded. In addition, a female of age 55 who said that she had no past history of brain injury but actually she had a significantly large brain atrophy due to a past history of severe brain injury, which was finally revealed by PET/CT, were excluded.

For dosimetry, dynamic scan of whole body of four volunteers showed that mean effective dose yielded 0.00848 ± 0.00125 mSv/MBq and 3.14 ± 0.46 mSv/370MBq. Measurement of blood and plasma radioactivity of metabolites showed no major metabolites (data not shown), which implied that [^18^F]FPYBF-2 was metabolically stable in vivo. No adverse events were reported during at least two-years follow-up period.

Average Mean Cortical Index of healthy volunteers at 50-70min after injection was calculated separately in each age range (20-39, 40-49, 50-59, 60-69, 70-79 years old; n= 8, 18, 16, 7, 12, respectively). Mean Cortical Index of them (1.024+/-0.045, 1.059+/-0.053, 1.062+/-0.064, 1.078+/-0.091, 1.096+/-0.093, respectively) were almost similar, except for the difference between the group 20-39yo and the group 70-79yo (p<0.05) **(**Suppl. **Fig. 1).**

Further evaluation of regional SUVR in each region of cortex was shown in Suppl. Fig. 2. SUVR of frontal, parietal lobe, cingulum and limbic lobe (in this text, it means the combined area of anterior, middle, and posterior cingulum, hippocampus, and para hippocampal area) did not show increase with advancing age. The 20-minutes static scan was separately calculated in two time-zone (50-60min and 60-70min), but the difference between these two time zone was small (1.057+/-0.067, 1.058+/-0.061, respectively).

^18^F-FPYBF-2 was mainly trapped in liver and its excretion was via urine and bile duct (Suppl. Fig. 3). No adverse events were reported during at least one-year follow-up period.

In addition, for the further evaluation of appropriate scan timing for the brain accumulation of ^18^F-FPYBF-2, additional 5hr-prolonged PET scan was also performed in one volunteer who gave informed consent to this prolonged scan, at 110-120min, 230-240min, 290-300min after injection of ^18^F-FPYBF-2, at the same instant (Suppl. Fig. 4). In this prolonged scan, Mean Cortical Index of SUVR reached plateau at 1 hour and showed stable up to 5 hours after the injection of ^18^F-FPYBF-2, which suggests that appropriate scan timing for ^18^F-FPYBF-2 is 1 hour after injection or later.

According to Table 1, 2 and Suppl. Fig. 4, it was clarified that appropriate scan time for the diagnosis of dementia would be around 1 hour or later after the injection of ^18^F-FPYBF-2 and only 10-minutes scan would be enough for diagnosis. Mean Cortical Index of SUVR reached plateau at 1 hour and showed stable up to 5 hours after the injection of ^18^F-FPYBF-2. This may suggest the in vivo equilibration of ^18^F-FPYBF-2 as an amyloid PET tracer. Mean Cortical Index of SUVR showed stable value both at 50-60 minutes scan and 60-70 minutes scan, therefore 10-minutes scan either at 50-60 minutes or 60-70 minutes would be adequate. This is because of the longer half-life of F-18 and the resultant higher retention of radioactivity of ^18^F-FPYBF-2 in brain, which results in a clear PET image with low noise. This clear PET image acquisition cannot be achieved by ^11^C-PiB PET/CT due to its shorter half-life of C-11. Shorter scan time of 10 minutes would be considered to be patient-friendly scan, which is appropriate for a patient with dementia. In this study, the merit of ^18^F-FPYBF-2 as a F-18 amyloid tracer was clearly elucidated.

***Results of Patients***

Average Mean Cortical Index of early stage and moderate stage AD patients were also significantly higher than that of other dementia patients (p<0.001, p<0.005). Average Mean Cortical Index of MCI patients was slightly lower than those of AD patients (p<0.05) and higher than that of other dementia patients (p<0.005).

Further evaluation of regional SUVR in each region of cortex was shown in Suppl. Fig. 5 and 6. Regional SUVR of frontal, parietal, occipital and temporal lobe showed similar pattern as to that of Mean Cortical Index as shown in Fig. 3. Regional SUVR of cingulum and limbic love were significantly higher than those of other area.

Evaluation of averaged Mean Cortical Index in two time-zone (50-60min and 60-70min) showed that the difference between these two time-zone was similar in total patients (1.9%) as compared to healthy volunteers (1.8%), while the difference was higher in both AD patients (2.0% in early AD and 2.4% in moderate stage AD). No adverse events were reported during at least one-year follow-up period.

**DISCUSSION**

Fig. 3 showed that differential diagnosis between AD patients and healthy volunteers was achieved by using the qualitative analysis of Mean Cortical Index of SUVR, and that the threshold of Mean Cortical Index was about 1.2. According to the threshold value 1.2 for ^18^F-FPYBF-2, the amyloid positive rates in whole healthy volunteers, younger healthy volunteers (49 yo or younger), older healthy volunteers (50 yo or older), much older healthy volunteers (60 yo or older), patients with MCI, and those with AD were follows; 4.9%, 0%, 8.6%, 10%, 31%, and 74%.

^18^F-AZD4694, known as another fluorinated benzofuran derivative, was evaluated with and without ^11^C-PiB [31,33]. Their results were quite comparable with our results of ^18^F-FPYBF-2, where their study shows similar TACs of arterial blood and each brain region, similar uptake pattern in axial brain PET images and the results of differential diagnosis between healthy volunteers and AD patients. However, 18F-AZD4694 showed roughly the same SUVR as 11C-PiB, presenting higher SUVR than 18F-FPYBF-2. Although ^18^F-AZD4694 and ^18^F-FPYBF-2 are different in the presence of a fluoropolyethylene glycol side chain and this difference might be the cause of the value of SUVR, their reports and our data may suggest the reliability of these fluorinated benzofuran derivatives as amyloid imaging tracers.

Of these ^18^F radiotracers, ^18^F-florbetapir (Amyvid), ^18^F-flutemetamol (Vizamyl) and ^18^F-florbetaben (Neuraceq) were already approved by the US Food and Drug Administration (FDA) and some of them are expected to be approved for health insurance coverage also in Japan in future. In such a clinical situation, we have to consider the role of ^18^F-FPYBF-2 in clinical practice and in research fields. By self-imposed criteria of academic societies, the appropriate use of the three amyloid imaging agents mentioned above may be strictly limited in a certain clinical condition, such as requirement of disease stage, qualification of referring doctor, etc [38,39]. In contrast, ^18^F-FPYBF-2 is independently-developed PET tracers of “all-made in Japan” from drug development to clinical practice without aids from large pharmaceutic developers [13], so ^18^F-FPYBF-2 would be simple to use and relatively easier to apply for research purpose. Especially in the research fields of long-term follow-up cohort study evaluating healthy volunteers and patients with Mild Cognitive Impairment, the use of ^18^F-FPYBF-2 would be easy to apply in large number of subjects because such kind of cohort study requires many subjects evaluated per one synthesis. In the present study, our capacity of synthesis of ^18^F-FPYBF-2 was up to more than 10 subjects per one synthesis because the average amount of produced ^18^F-FPYBF-2 was about 14 GBq at every synthetic day in our study. With the 10-minute static scan, ^18^F-FPYBF-2 PET/CT may accelerate the fast and efficient inspection with larger number of subject in cohort studies. Further utilization of ^18^F-FPYBF-2 for a tracer of cohort study of healthy volunteers would be needed.

**Reference**

38.

Johnson KA, Minoshima S, Bohnen NI, Donohoe KJ, Foster NL, Herscovitch P, et al; Amyloid Imaging Task Force of the Alzheimer’s Association and Society for Nuclear Medicine and Molecular Imaging. Update on appropriate use criteria for amyloid PET imaging: dementia experts, mild cognitive impairment, and education. Amyloid Imaging Task Force of the Alzheimer’s Association and Society for Nuclear Medicine and Molecular Imaging. Alzheimers Dement. 2013;9:e106-9.

39.

Johnson KA, Minoshima S, Bohnen NI, Donohoe KJ, Foster NL, Herscovitch P, et al. Update on appropriate use criteria for amyloid PET imaging: dementia experts, mild cognitive impairment, and education. J Nucl Med. 2013;54:1011-3.

**Figure Legends**

**Supplementary Figure 1.**

Results of Mean Cortical Index of SUVR in healthy volunteers. Each group represents a volunteer group with 20-39 y.o., 40-49 y.o., 50-59 y.o., 60-69 y.o. and 70-79 y.o.. There was slight increase in Mean Cortical Index with advancing age (1.024+/-0.045, 1.059+/-0.053, 1.062+/-0.064, 1.078+/-0.091, 1.096+/-0.093, respectively). However, the difference of Mean Cortical Index in each age range was not significant, except for the difference between the group 20-39yo and the group 70-79yo (p<0.05).

**Supplementary Figure 2.**

Regional SUVR in each region of cortex at healthy volunteer study was shown. SUVR of frontal, parietal lobe, cingulum and limbic lobe did not show increase with advancing age. Please note that the SUVR of cingulum and limbic lobe were higher than that of frontal, parietal, occipital and temporal lobe (not shown) at healthy volunteer study. In this text, “limbic lobe” means the combined area of anterior, middle, and posterior cingulum, hippocampus, and para hippocampal area.

**Supplementary Figure 3.**

Maximum Intensity Projection (MIP) image of whole body scan (front view) of ^18^F-FPYBF-2 one hour after IV injection in a healthy volunteer, 50 y.o., male. Slight uptake in brain can be observed at the top of whole body. Prominent uptake in the liver, small intestine and urinary bladder suggests that the excretion route of this agent is bile and urine. Please note the retention of ^18^F-FPYBF-2 in the left upper arm which was near to the injection site (left elbow). It implies that ^18^F-FPYBF-2 has viscid and adhesive property.

**Supplementary Figure 4.**

Time course of radioactivity of each region of brain at 5-hours prolonged scan of ^18^F-FPYBF-2 PET performed in a patient. Mean Cortical Index of SUVR reached plateau at 1 hour and showed stable up to 5 hours after the injection of ^18^F-FPYBF-2, which suggests that appropriate scan timing for ^18^F-FPYBF-2 is 1 hour or later.

**Supplementary Figure 5.**

Regional SUVRs of frontal, parietal, occipital and temporal lobe of various patients and volunteers were shown. In these areas, similar pattern was observed as to that of Mean Cortical Index shown in Fig. 3. Statistical analysis for all groups was performed by Kruskal-Wallis Analysis for unpaired data.

**Supplementary Figure 6.**

Regional SUVR of cingulum, limbic lobe, thalamus and subcortical area of various patients and volunteers were shown. Statistical analysis for all groups was performed by Kruskal-Wallis Analysis for unpaired data, which showed a significant difference between each group. Please note that the SUVR of cingulum and limbic lobe were higher than that of frontal, parietal, occipital and temporal lobe in all groups. This is similar pattern to that observed at healthy volunteer study. Regional SUVRs of thalamus were higher in healthy volunteer group with younger age (59 y.o. or younger). However, there was no correlation between each disease group. The reason of this phenomenon is unknown.
